# Extracellular potassium concentration defines neuronal bursting properties

**DOI:** 10.1186/1471-2202-16-S1-P215

**Published:** 2015-12-18

**Authors:** Yaroslav I Molkov, Bartholomew J Bacak, Joshua Segaran, Ilya A Rybak

**Affiliations:** 1 Department of Mathematical Sciences, Indiana University-Purdue University, IN, USA; 2 Department of Neurobiology and Anatomy, Drexel University College of Medicine, PA 19123, USA; 3 Carmel High School, Carmel, IN 46032, USA

## 

Many neurons, or populations of neurons, in the brain are capable of producing rhythmic bursting activity. This ability is putatively responsible for rhythmogenic functions like breathing and locomotion. *In vivo*, rhythms are generated by synaptically interconnected neuronal networks, whereas rhythmic bursting behavior is often induced *in vitro *by elevating the extracellular potassium concentration (*K_out_*) [1]. It is known that increasing *K_out _*raises the reversal potentials of potassium and leak currents [2]. However, the complete nature of how these effects underlie bursting activity has yet to be uncovered.

A mathematical modeling study was performed to elucidate the interplay between these factors and their roles in a neuron's transition from quiescence to rhythmic bursting. A conductance-based model of a neuron from the pre-Bötzinger Complex (pre-BötC) was used as a basis [3]. A potassium ion component was incorporated into the leak current, and model behaviors were investigated at varying concentrations of *K_out_*, taking into account its effect on delayed rectifier potassium current responsible for after-spike hyperpolarization. The primary aim of this modeling study was to evaluate the contribution of extracellular potassium ions in the leak and delayed rectifier potassium current, and the subsequent effect of these altered currents on the bursting properties of neurons. Furthermore, the initial model was modified to replicate experimental results and test for conditions of low *K_out _*as seen *in vivo*.

The analysis of our model shows that: (i) *in vitro *bursting behavior with elevated *K_out _*may occur due to attenuation of the delayed rectifier potassium current and (ii) no oscillations are generated at physiological levels of extracellular potassium. These results indicate that, according to the commonly-accepted models used in our study, neurons that naturally burst in *in vitro *preparations may not be able to burst *in vivo *under any circumstances. Accordingly, rhythmic activity *in vivo *should rely on other mechanisms. For example, Jasinski et al. [4] have shown that the recurrent synaptic excitation in combination with the sodium-potassium exchanger (pump) can result in the robust rhythmic network activity even with all intrinsic bursting mechanisms blocked.

## References

[B1] Smith JC Ellenberger HH Ballanyi K Richter DW Feldman JL Pre-Botzinger Complex - a Brain-Stem Region That May Generate Respiratory Rhythm in Mammals Science 1991 254 5032 726 729 1683005 10.1126/science.1683005 PMC3209964

[B2] Rybak IA Shevtsova NA St-John WM Paton JFR Pierrefiche O Endogenous rhythm generation in the pre-Botzinger complex and ionic currents: modelling and in vitro studies Eur J Neurosci 2003 18 2 239 257 12887406 10.1046/j.1460-9568.2003.02739.x

[B3] Butera RJ Rinzel J Smith JC Models of respiratory rhythm generation in the pre-Botzinger complex. I. Bursting pacemaker neurons J Neurophysiol 1999 82 1 382 397 10400966 10.1152/jn.1999.82.1.382

[B4] Jasinski PE Molkov YI Shevtsova NA Smith JC Rybak IA Sodium and calcium mechanisms of rhythmic bursting in excitatory neural networks of the pre-Botzinger complex: a computational modelling study Eur J Neurosci 2013 37 2 212 230 23121313 10.1111/ejn.12042 PMC3659238

